# Supplementary material for: A BAFF ligand-based CAR-T cell targeting three receptors and multiple B cell cancers
Source: Nat Commun. 2022 Jan 11;13:217. doi: 10.1038/s41467-021-27853-w (PMC8752722; doi:10.1038/s41467-021-27853-w)
Supplement: Supplementary file 1 — Supplementary Information [file 41467_2021_27853_MOESM1_ESM.pdf]

Supplementary Figure 1

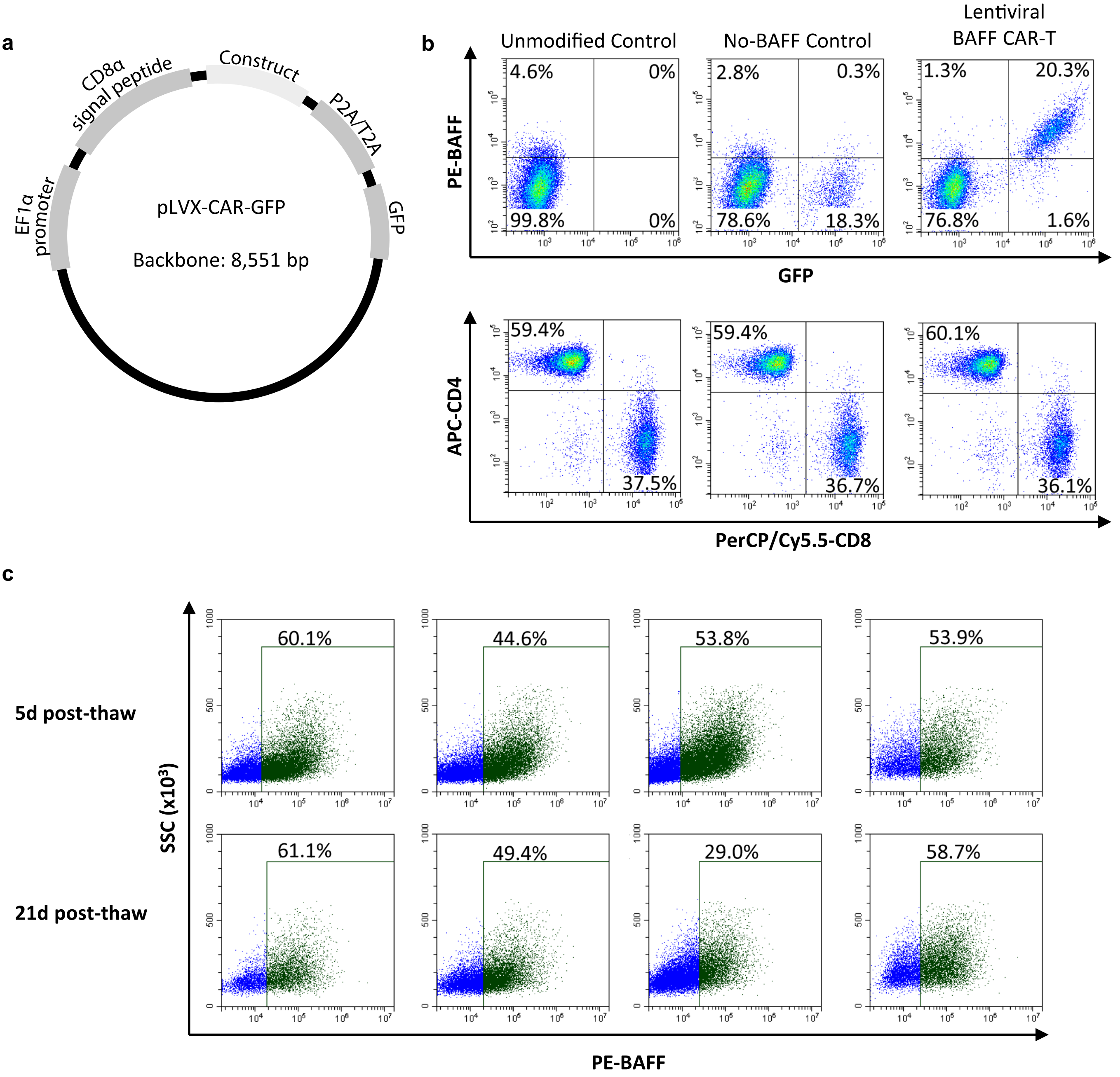

Supplementary Figure 1. A custom lentiviral vector was used to transduce T cells and express the BAFF-CAR construct, a) Map of the custom pLVX lentiviral vector. Expression is driven by the EF1α promoter. A signal peptide derived from the leader sequence of CD8α is used to direct the construct to the cell surface. GFP is expressed and cleaved from the construct downstream of a tandem P2A/T2A self-cleaving peptide sequence. **b)** BAFF-CAR and no-BAFF control lentiviral transduction efficiency in primary human T cells was measured 5 days after transduction. No-BAFF control T cells express the truncated construct that lacks BAFF ligand but still expresses GFP. **c)** Stability of BAFF CAR-T cells generated using the *TcB* transposon system was assessed by freezing T cells 7d after transfection, then thawing them and comparing cell surface BAFF expression 5 days post-thaw and 21 days post-thaw. Data are displayed for 4 different donors.

Supplementary Figure 2

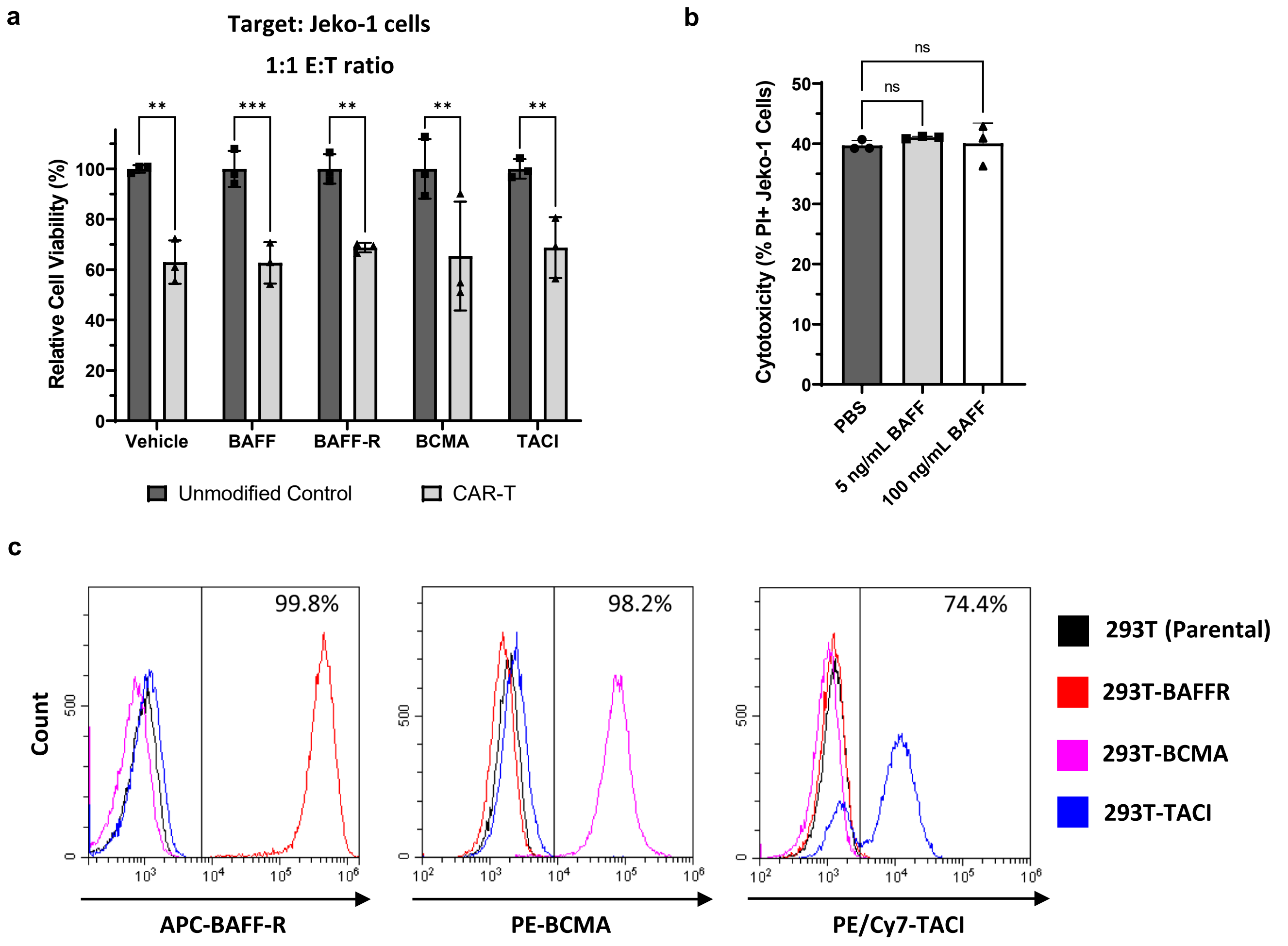

Supplementary Figure 2. Functionality and specificity of the BAFF-CAR construct were assessed using different experimental methods. a) CAR-T cells or unmodified T cells were co-cultured with luciferase-expressing Jeko-1 cells at 1:1 E:T ratio for 24 h in the presence of soluble recombinant BAFF or BAFF receptors to determine whether the presence of BAFF or BAFF receptors in co-culture media interferes with BAFF-CAR function. The following concentrations of soluble protein were used: BAFF = 1 ng/mL; BAFF-R = 500 ng/mL; BCMA = 2.5 ng/mL; TACI = 20 pg/mL. Vehicle served as a negative control. Luminescence was measured using a plate reader. % viability was calculated using target-only control, then normalized relative to average luminescence from target cells co-cultured with unmodified T cells. **P<0.01, ***P<0.001. The following comparisons are between Unmodified Control and CAR-T: P=0.001 for Vehicle, P=0.0009 for BAFF, P=0.0052 for BAFF-R, P=0.0020 for BCMA, P=0.0053 for TACI. Mean±SD, n=3 biologically independent co-cultures, 2-way ANOVA with Šídák's multiple comparison test. Experiment was repeated with 3 different T cell donors. **b)** CAR-T cells, transduced control T cells, or unmodified T cells were co-cultured with fluorescently-labeled Jeko-1 cells at 5:1 E:T ratio for 24 h in the presence of different concentrations of soluble recombinant human BAFF at a pathophysiological concentration (5 ng/mL) or a non-physiological very high concentration (100 ng/mL) to determine whether the presence of BAFF interferes with BAFF-CAR function. ns = not significant. Mean±SD, n=3 biologically independent co-cultures, 1-way ANOVA with Dunnett's multiple comparisons test. Experiment was repeated with 2 different T cell donors. **c)** Flow cytometry was used to confirm HEK293T exogenous expression of BAFF-R, BCMA, or TACI. These cells were used in Fig. 3b to further evaluate BAFF-CAR specificity. Black = parental HEK293T; red = BAFF-R-expressing HEK293T; purple = BCMA-expressing HEK293T; blue = TACI-expressing HEK293T. Source data for all graphs are provided as a Source Data file.

Supplementary Figure 3

a

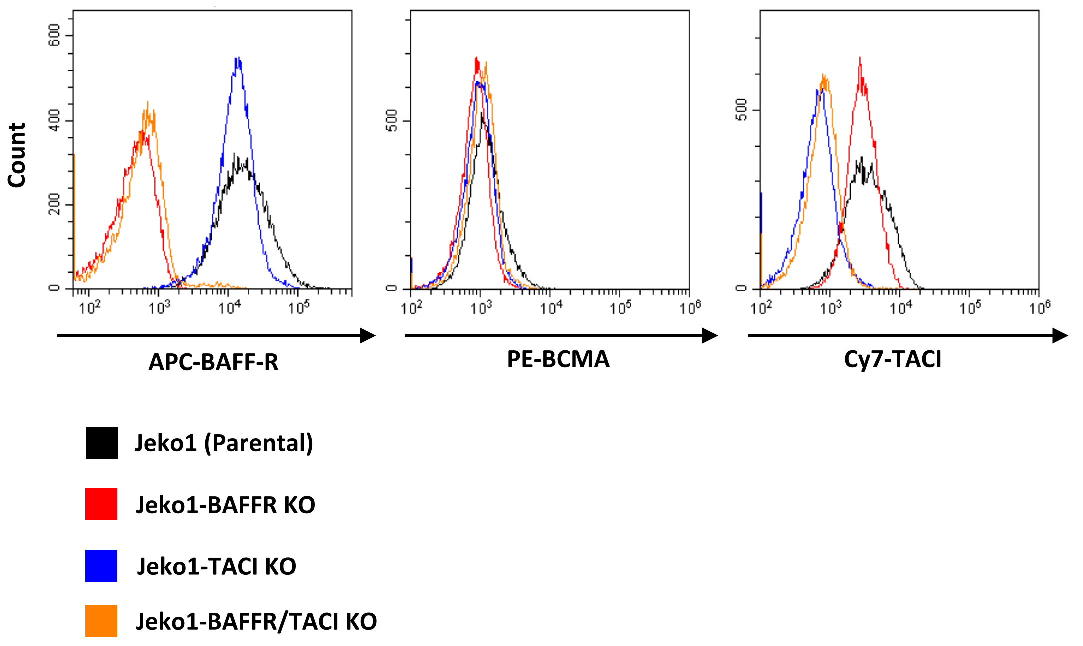

b

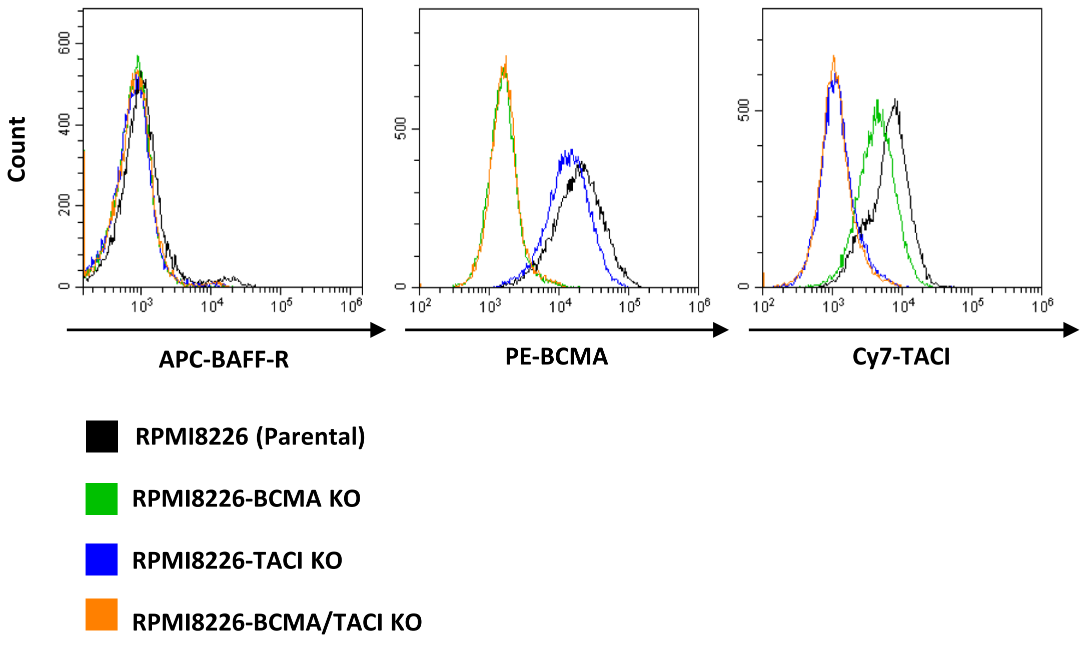

Supplementary Figure 3. Confirmation of CRISPR knockout of the BAFF receptors in Jeko-1 and RPMI-8226 cells. a) Flow cytometry was used to confirm CRISPR-mediated single or dual knockout of BAFF-R and TACI receptors in Jeko-1 cells. Black = parental Jeko-1; red = BAFF-R knockout Jeko-1; blue = TACI knockout Jeko-1; orange = dual BAFF-R/TACI knockout Jeko-1. **b)** Flow cytometry was used to confirm CRISPR-mediated single or dual knockout of BCMA and TACI receptors in RPMI-8226 cells. Black = parental RPMI-8226; green = BCMA knockout RPMI-8226; blue = TACI knockout RPMI-8226; orange = dual BCMA/TACI knockout RPMI-8226.

Supplementary Figure 4

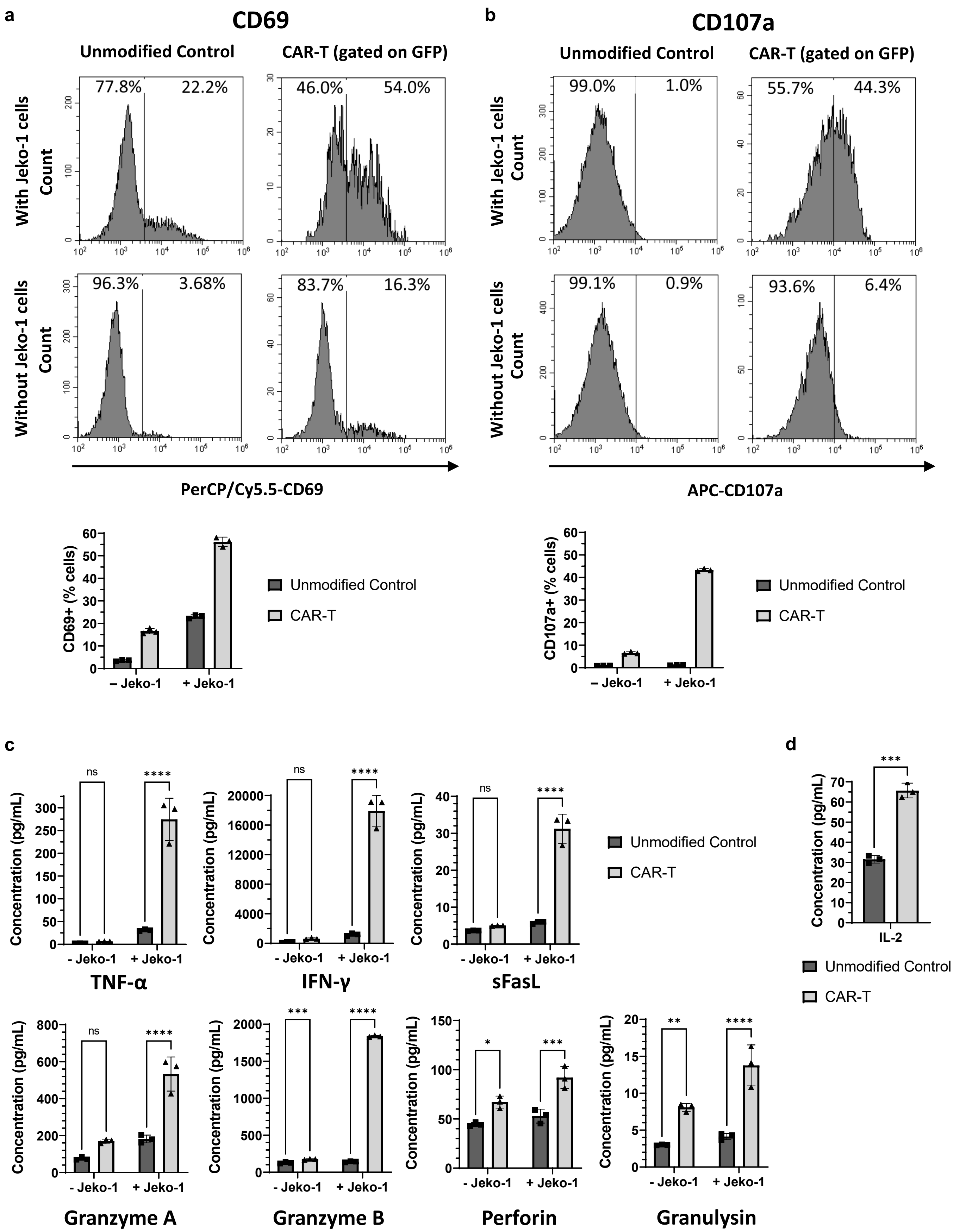

Supplementary Figure 4. Baseline cytokine release, CD69, and CD107a expression of BAFF CAR-T cells. a) T cells were cultured alone or co-cultured with labeled Jeko-1 cells at 5:1 E:T ratio for 24 h, then stained for CD69. % CD69+ T cells were measured via flow cytometry after gating on live cells and excluding labeled cancer cells; for CAR-T samples, additional gating over GFP+ cells was applied to exclude unmodified cells. Flow histograms display how CD69+ percentage was measured. Mean±SD, n=3 biologically independent co-cultures. Experiment was repeated with 2 different T cell donors. **b)** T cells were cultured alone or co-cultured with Jeko-1 cells at 5:1 E:T ratio for 6 h while staining for the degranulation marker CD107a. % CD107a+ T cells were measured via flow cytometry after gating on CD3+ cells; for CAR-T samples, additional gating over GFP+ cells was applied to exclude unmodified cells. Flow histograms display how CD107a+ percentage was measured. Mean±SD, n=3 biologically independent co-cultures. Experiment was repeated with 2 different T cell donors. **c)** T cells were cultured alone or co-cultured with Jeko-1 cells at 1:1 E:T ratio for 16h. Culture supernatant was collected to measure T cell release of pro-inflammatory cytokines and lytic enzymes. ns = not significant, *P<0.05, **P<0.01, ***P<0.001, ****P<0.0001. The following p-values are listed for statistically significant comparisons between Unmodified Control and CAR-T, -Jeko-1 cells: Granzyme B: P=0.0004; Perforin: P=0.011; Granulysin: P=0.0047. The following p-values are listed for statistically significant comparisons between Unmodified Control and CAR-T, + Jeko-1 cells: TNF-α: P<0.0001; IFN-γ: P<0.0001; sFasL: P<0.0001; Granzyme A: P<0.0001; Granzyme B: P<0.0001; Perforin: P=0.0004; Granulysin: P<0.0001. Mean±SD, n=3 biologically independent co-culture samples, 2-way ANOVA with Šídák's multiple comparisons test. Experiment was repeated with 2 different T cell donors. **d)** T cells and Jeko-1 cells were co-cultured in IL-2-free media to measure T cell secretion of IL-2. ***P<0.001. P=0.0001 for Unmodified Control vs CAR-T. Mean±SD, n=3 biologically independent co-culture samples, unpaired two-tailed t-test. Source data for all graphs are provided as a Source Data file. Experiment was repeated with 2 different T cell donors.

Supplementary Figure 5

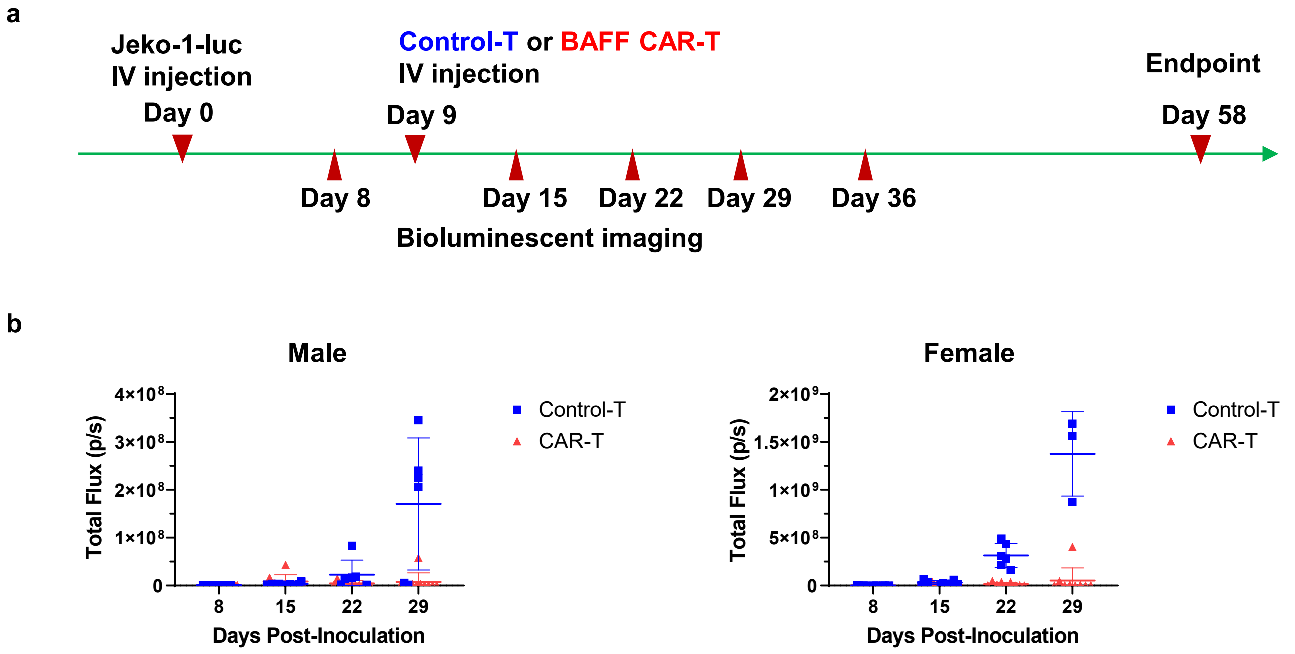

Supplementary Figure 5. Schematic and bioluminescence quantification data for i.v. MCL xenograft model. a) Schematic for intravenous MCL xenograft experiment. NSG mice were injected i.v. via tail vein with 1.5e6 Jeko-1-luc cells on Day 0. On Day 9, 10e6 BAFF CAR-T cells or Control-T cells were injected i.v. Bioluminescence imaging was performed weekly up to Day 36, with the experiment continuing until Day 58. **b)** Total flux (photons/s) is plotted over 36 days post-inoculation using measurements from 4 days of imaging. Blue = Control-T, red = CAR-T. Mean±SD, n=9 mice per treatment group. Source data for all graphs are provided as a Source Data file. Experiment was repeated with 2 different T cell donors.

Supplementary Figure 6

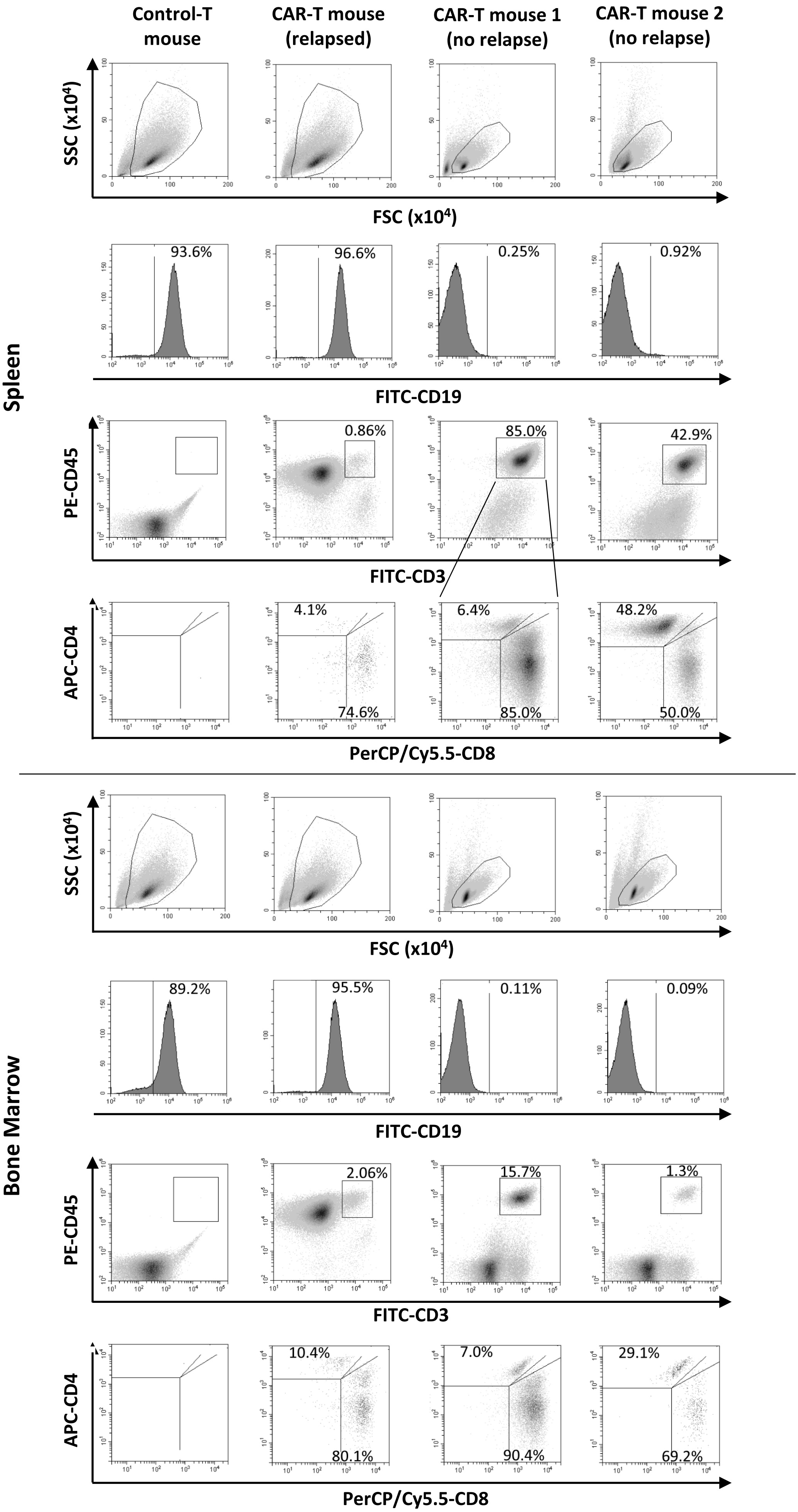

Supplementary Figure 6. Jeko-1 cells in spleen and bone marrow of treated mice. Spleen and bone marrow were collected from mice at time of euthanasia in the i.v. Jeko-1 experiment. After red blood cell lysis, white blood cells were stained for human CD19 or human CD3 and CD45 to detect whether Jeko-1 cells or T cells were present in these compartments. Representative flow plots and histograms from a Control-T-treated mouse, a CAR-T-treated mouse displaying cancer relapse, and two CAR-T-treated mice without detectable tumor burden are shown. Histograms display CD19+ Jeko-1 cell percentage out of total WBCs. Flow plots display CD3+/CD45+ T cell percentage out of total WBCs, as well as CD4+ and CD8+ T cell percentages out of gated T cells.

Supplementary Figure 7

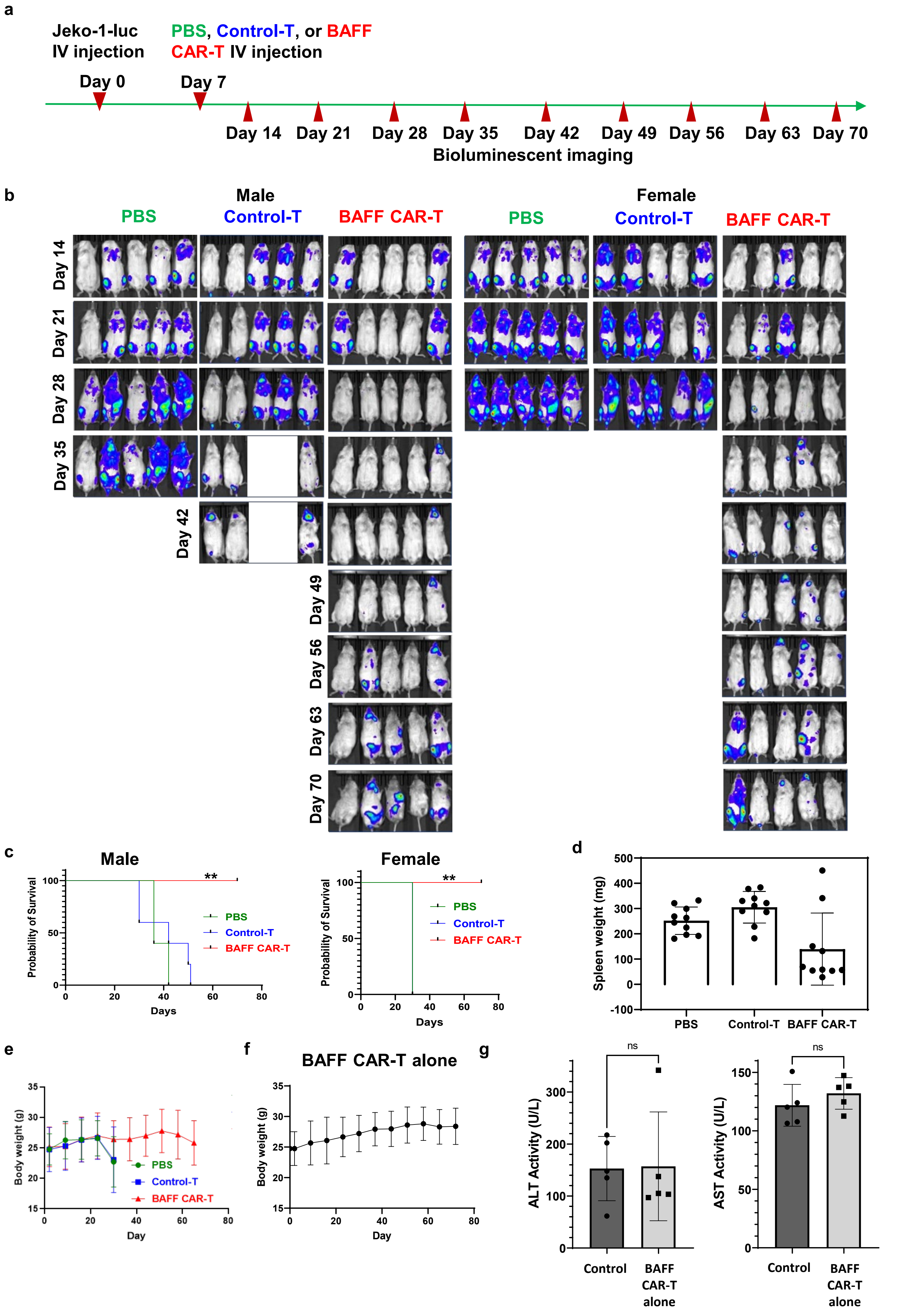

Supplementary Figure 7. A lower dose of BAFF CAR-T cells still performs well in an i.v. Jeko-1 MCL model. a) Schematic for intravenous MCL xenograft experiment with lower-dose treatment. NSG mice were injected i.v. via tail vein with 1.5e6 Jeko-1-luc cells on Day 0. On Day 7, 3e6 BAFF CAR-T cells, Control-T cells, or PBS alone were injected i.v. **b)** Bioluminescence imaging of mice commenced 14 days post-inoculation (7 days post-treatment) and continued weekly up to Day 70. Quantification of bioluminescence signal intensity was determined using the Living Image software (Perkin Elmer). Black = PBS, blue = Control-T, red = BAFF CAR-T. n=5 mice per treatment group. **c)** Kaplan-Meier survival curves for male and female mice from the 3 different treatment groups. **$P<0.01$. For male mice: $P=0.0051$ for PBS vs BAFF CAR-T and Control-T vs BAFF CAR-T. For female mice: $P=0.0081$ for PBS vs BAFF CAR-T and Control-T vs BAFF CAR-T. n=5 mice per treatment group. Log-rank (Mantel-Cox) tests were applied, followed by Holm-Šídák correction for multiple comparisons. **d)** Summary of mouse spleen weight from separate treatment groups at time of sacrifice. Male and female mouse spleens were pooled together within each treatment group (n=10 spleens per treatment group). Mean±SD. **e)** Pooled body weight measurements of both male and female mice from the three treatment groups throughout the study interval (n=10 mice per treatment group). Mean±SD. **f)** Pooled body weight measurements of both male and female mice injected with BAFF CAR-T cells alone, without tumor (n=10 mice). Mice were tracked over a 70-day period. Mean±SD. **g)** Serum from healthy untreated mice (Control) and healthy mice injected with 3e6 BAFF CAR-T cells alone, both without tumor inoculation, were collected on 70 days post-injection. Serum alanine aminotransferase (ALT) and aspartate aminotransferase (AST) activity were assayed to assess liver enzyme levels in circulation. ns = not significant. Mean +/- SD, n=5 mice, 3 technical triplicates per serum sample, unpaired two-tailed t-test. Source data for all graphs are provided as a Source Data file. Experiment was repeated with 2 different T cell donors.

Supplementary Figure 8

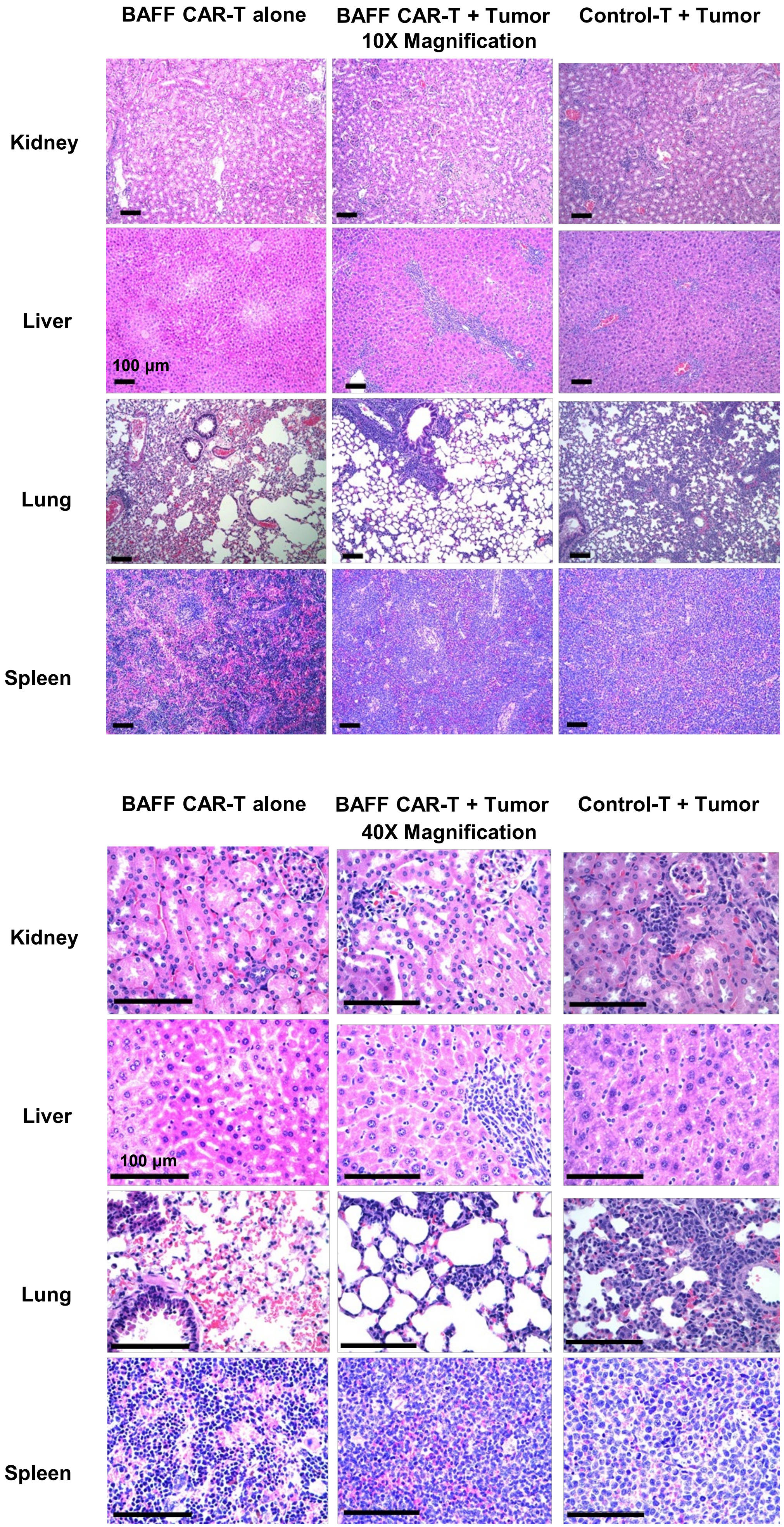

Supplementary Figure 8. Histology shows no significant tissue damage from BAFF CAR-T treatment in i.v. Jeko-1 MCL model. Histological sections of tissues from mice treated with BAFF CAR-T cells alone without tumor, BAFF CAR-T + Tumor, and Control-T + Tumor are shown. Kidney, liver, lung, and spleen were harvested from euthanized mice 30-75 days post-inoculation, and tissue sections were stained with hematoxylin and eosin. No lesions were apparent and no significant tissue damage was noted in BAFF CAR-T injected mice compared to Control-T cell injected mice. Normal architecture was maintained and there were no obvious, significant differences in the structures of the kidney, liver, lung, and spleen between the BAFF CAR-T-treated and Control-T-treated groups (scale bar = 100 μm, 10X and 40X magnifications are shown). Histological sections were obtained from n=3 mice per treatment group in the experiment described in Supplementary Fig. 7. Displayed images are representative of mice from each treatment group.

Supplementary Figure 9

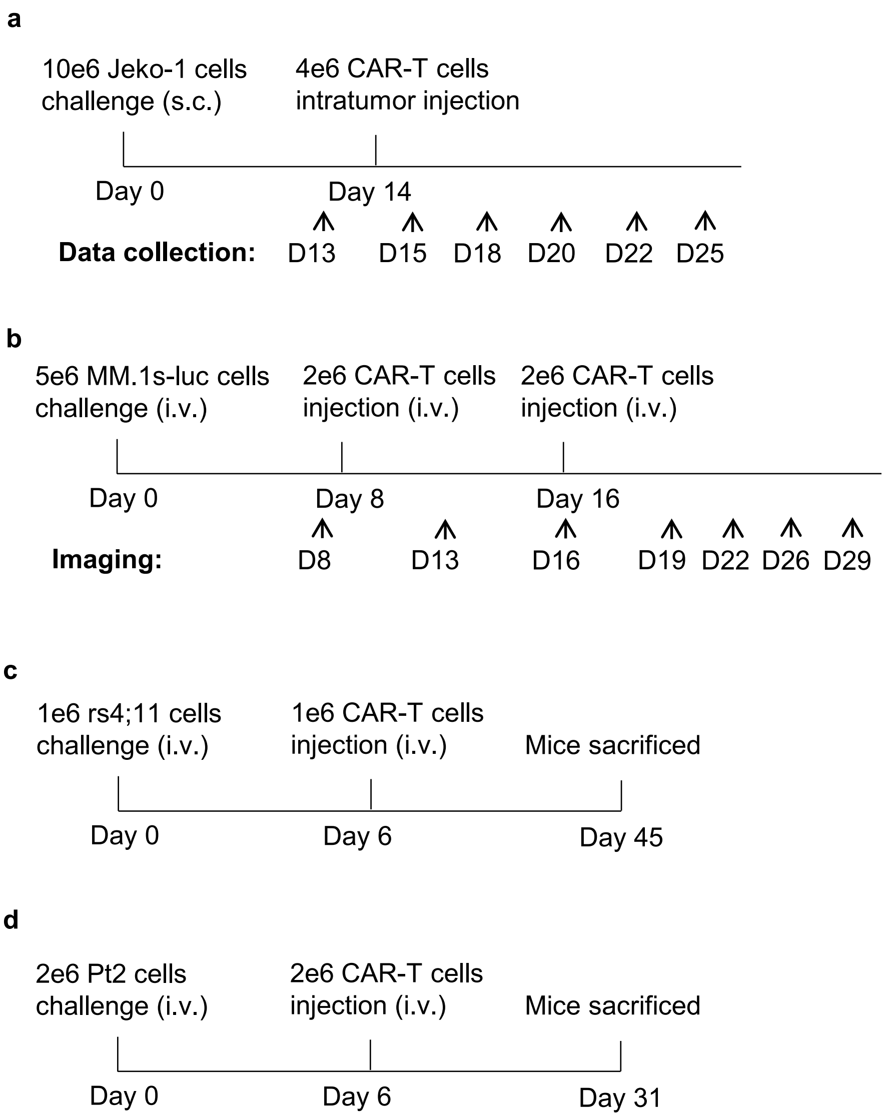

Supplementary Figure 9. Schematics for s.c. MCL, i.v. MM, and i.v. ALL xenograft models. a) 10e6 Jeko-1 cells were injected into NSG mice subcutaneously above the flank on Day 0. After palpable tumor formation, 4e6 CAR-T cells, unmodified Control-T cells, or PBS alone were injected intratumorally on Day 14. Tumor width and length were measured every 2-3 days, as indicated. **b)** 5e6 MM.1s cells were injected into NSG mice intravenously via tail vein on Day 0. 2e6 CAR-T cells or unmodified Control-T cells were injected i.v. on Day 8 and Day 16 after tumor implantation was confirmed. Bioluminescence imaging was continued up to Day 29 post-inoculation. **c)** 1e6 rs4;11 cells were injected into NSG mice intravenously via tail vein on Day 0. 1e6 CAR-T cells or PBS alone were injected via tail vein on Day 6. Mice were sacrificed at the endpoint, Day 45 post-inoculation. **d)** 2e6 Patient ALL (Pt2) cells were injected into NSG mice intravenously via tail vein on Day 0. 2e6 CAR-T cells or PBS alone were injected via tail vein on Day 6. Mice were sacrificed at the endpoint, Day 31 post-inoculation.

Supplementary Figure 10

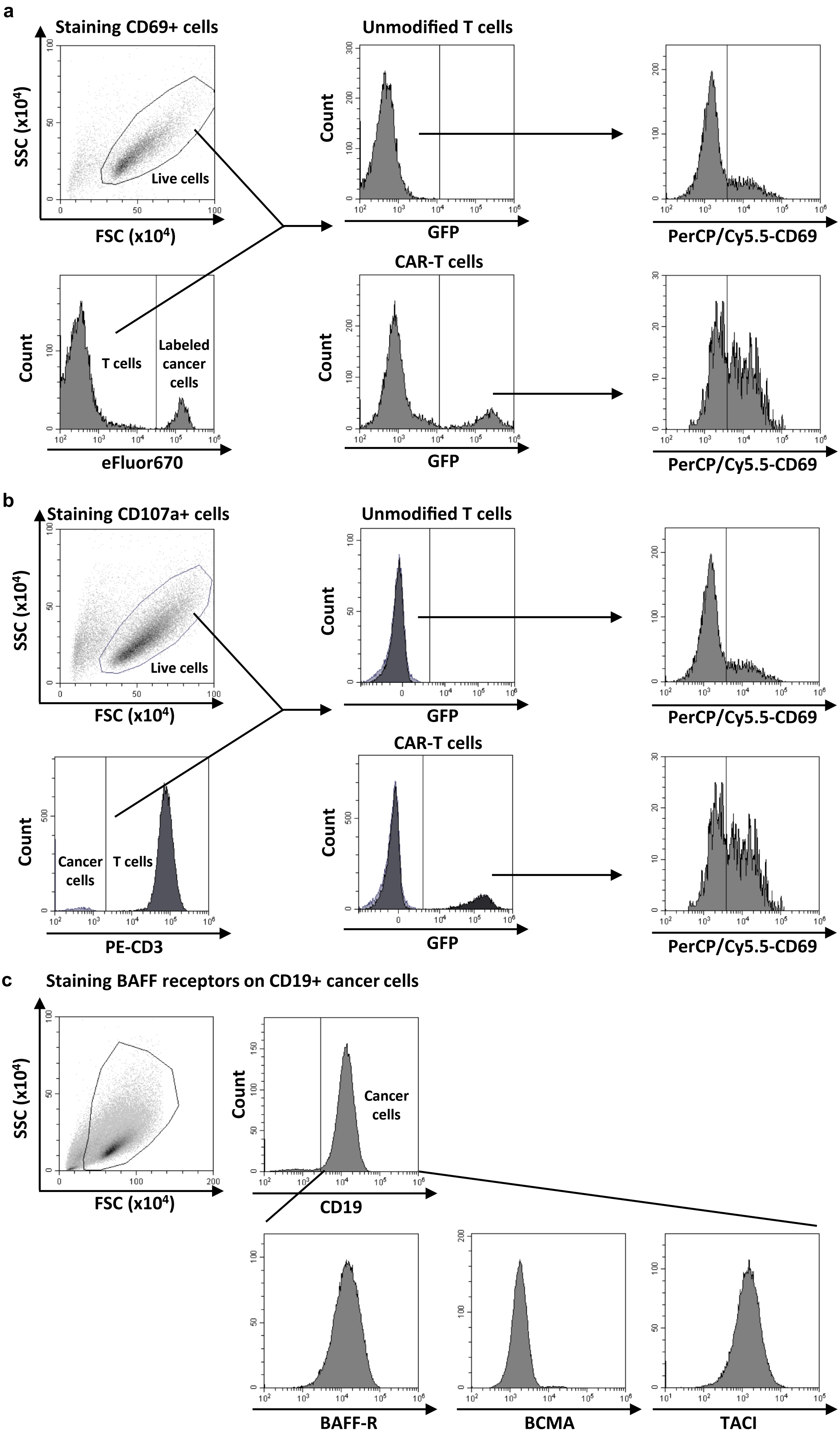

Supplementary Figure 10. Flow cytometry gating strategies. a) CD69+ T cell staining strategy. Labeled tumor cells were excluded and BAFF CAR-T samples were gated on live GFP+ cells, while unmodified T cell samples were gated on live cells. CD69 histograms correspond to panels in Supplementary Fig. 4a. **b)** CD107a+ T cell staining strategy. BAFF CAR-T samples were gated on live CD3+ GFP+ cells, while unmodified T cell samples were gated on live CD3+ cells. CD107a histograms correspond to panels in Supplementary Fig. 4b. **c)** BAFF receptor staining on tumor cells from xenograft models. Single cell suspensions from mouse spleen and bone marrow were gated on CD19+ tumor cells. BAFF-R, BCMA, and TACI histograms correspond to panels in Fig. 6e.

Supplementary Table 1

Name	Total unique insertion sites	Insertion sites not in transcript (%)	Insertion sites within transcript (%)	Insertion sites within intron (%)	Insertion sites within exon (%)	Insertion sites within coding exon (%)	Median distance to transcriptional start site	Most abundant clone reads %	Top 10 most abundant clone reads %
TcB-M Donor 1	14,931	41.8%	58.2%	54.4%	6.3%	1.1%	19.6	0.05%	0.4%
TcB-M Donor 10	7,382	38.3%	61.7%	57.7%	6.6%	0.7%	17.3	0.08%	0.7%
TcB-M Donor 11	14,364	38.4%	61.6%	57.3%	7.1%	1.3%	17.2	0.05%	0.4%
TcB-M Donor 13	20,781	41.3%	58.7%	54.9%	6.3%	1.1%	19.6	0.04%	0.4%
TcB-M Donor 15	37,997	40.5%	59.5%	56.1%	5.7%	1.1%	19.8	0.03%	0.2%
TcB-M Donor 16	8,974	39.2%	60.8%	56.7%	6.6%	1.0%	17.6	0.07%	0.6%
TcB-M Donor 17	18,773	39.7%	60.3%	56.4%	6.4%	1.0%	18.7	0.04%	0.4%
TcB-M Donor 18	31,417	40.5%	59.5%	55.7%	6.4%	1.2%	18.4	0.02%	0.2%
Transposon Only Donor 1	416	49.8%	50.2%	49.0%	2.4%	1.0%	28.0	1.19%	9.9%
Transposon Only Donor 10	30	46.7%	53.3%	53.3%	0.0%	0.0%	14.7	8.06%	59.2%
Transposon Only Donor 11	21	42.9%	57.1%	57.1%	0.0%	0.0%	24.6	11.81%	74.8%
Transposon Only Donor 13	212	43.9%	56.1%	54.7%	1.9%	0.0%	28.2	1.93%	16.9%
Transposon Only Donor 15	4	50.0%	50.0%	50.0%	25.0%	0.0%	73.0	47.06%	100.0%
Transposon Only Donor 16	67	49.3%	50.7%	47.8%	3.0%	0.0%	20.0	10.67%	54.2%
Transposon Only Donor 17	39	64.1%	35.9%	30.8%	5.1%	0.0%	61.3	8.36%	58.2%
Transposon Only Donor 18	83	50.6%	49.4%	43.4%	6.0%	1.2%	36.2	6.77%	39.3%
PiggyBac	22,620 ±21,456	42.20% ±3.52%	57.80% ±3.52%	55.78% ±2.83%	3.69% ±1.77%	0.36% ±0.28%	23.6 ±6.0	2.52% ±1.74%	9.12% ±2.83%
Sleeping Beauty	34,213 ±20,317	49.61% ±4.46%	50.39% ±4.46%	49.40% ±4.14%	1.68% ±0.63%	0.14% ±0.06%	33.8 ±5.2	2.58% ±1.37%	13.80% ±7.76%
Lentivirus (Wang)	27,482 ±990	15.24% ±0.19%	84.76% ±0.19%	79.92% ±0.10%	7.75% ±0.12%	3.05% ±0.23%	14.0 ±0.3	0.50% ±0.25%	0.96% ±0.34%
Lentivirus (Brady)	273 ±51	14.72% ±1.20%	85.28% ±1.20%	81.16% ±1.89%	8.15% ±3.27%	3.18% ±1.32%	11.2 ±2.1	2.17% ±0.56%	12.96% ±1.98%
Random (in silico)		46.38% ±0.18%	53.62% ±0.18%	51.02% ±0.19%	4.24% ±0.08%	1.25% ±0.04%	30.0 ±0.1		

Supplementary Table 1 – TcB integration site analysis for individual T cell donors. Total unique insertion sites, location of insertion sites, and median distance to transcriptional start sites are listed for each individual donor T cells that were transfected with both *TcB* transposase RNA and the BAFF-CAR transposon plasmid or with the transposon only. Data for *PiggyBac* and *Sleeping Beauty* transposons[1], Wang *et al.* and Brady *et al.* lentiviral transduction datasets[2,3], and random (in silico) integration values are also listed for comparison.

Supplementary Table 2

Antibody	Catalog #	Clone
BAFF (PE, anti-human)	318605	T7-241
BAFF-R (APC, anti-human)	316916	11C1
BCMA (PE, anti-human)	357504	19F2
TACI (PE/Cy7, anti-human)	311907	1A1
CD19 (FITC, anti-human)	392508	4G7
CD4 (APC, anti-human)	357408	A161A1
CD8 (PerCP/Cy5.5, anti-human)	344708	SK1
CD3 (FITC, anti-human)	300306	HIT3a
CD3 (PE, anti-human)	300308	HIT3a
CD45 (PE, anti-human)	368510	2D1
CD69 (PerCP/Cy5.5, anti-human)	310926	FN50
CD107a (APC, anti-human)	328619	H4A3

Supplementary Table 2 – Flow cytometry antibodies. Flow cytometry antibodies were obtained from Biolegend and used at 1/50 dilution.

Supplementary References

1 Gogol-Doring, A. *et al.* Genome-wide Profiling Reveals Remarkable Parallels Between Insertion Site Selection Properties of the MLV Retrovirus and the piggyBac Transposon in Primary Human CD4(+) T Cells. *Mol Ther* **24**, 592-606, doi:10.1038/mt.2016.11 (2016).

2 Wang, G. P., Ciuffi, A., Leipzig, J., Berry, C. C. & Bushman, F. D. HIV integration site selection: analysis by massively parallel pyrosequencing reveals association with epigenetic modifications. *Genome Res* **17**, 1186-1194, doi:10.1101/gr.6286907 (2007).

3 Brady, T. *et al.* HIV integration site distributions in resting and activated CD4+ T cells infected in culture. *AIDS* **23**, 1461-1471, doi:10.1097/QAD.0b013e32832caf28 (2009).
